# Risk Assessment of Hepatocellular Carcinoma Using Transient Elastography Vs. Liver Biopsy in Chronic Hepatitis B Patients Receiving Antiviral Therapy

**DOI:** 10.1097/MD.0000000000002985

**Published:** 2016-03-25

**Authors:** Yeon Seok Seo, Mi Na Kim, Seung Up Kim, Sang Gyune Kim, Soon Ho Um, Kwang-Hyub Han, Young Seok Kim

**Affiliations:** From the Division of Gastroenterology and Hepatology (YSS, SHU), Department of Internal Medicine, Institute of Digestive Disease and Nutrition, Korea University College of Medicine, Seoul; Department of Internal Medicine (MNK), CHA Bundang Medical Center, CHA University, Seongnam; Department of Internal Medicine (SUK, KHH), Institute of Gastroenterology, Yonsei University College of Medicine, Seoul; Department of Internal Medicine (SGK, YSK), Soonchunhyang University Bucheon Hospital, Bucheon; and Liver Cirrhosis Clinical Research Center (YSS, MNK, SUK, SGK, SHU, KHH, YSK), Seoul, Korea; The Korean Transient Elastography Study Group.

## Abstract

Liver stiffness (LS) assessed using transient elastography (TE) can assess the risk of developing hepatocellular carcinoma (HCC). We evaluated whether TE, when compared with histological data as a reference standard, can predict the risk of HCC development in chronic hepatitis B (CHB) patients starting antiviral therapy.

Observational cohort database of 381 patients with CHB who underwent liver biopsy (LB) and TE were reviewed. All patients underwent surveillance for HCC development using ultrasonography and alpha-fetoprotein.

During the median follow-up period of 48.1 (interquartile range 30.3–69.3) months, HCC developed in 34 (8.9%) patients. In patients with HCC development, age, proportion of diabetes mellitus, histological fibrosis stage, and LS value were significantly higher than those in patients without (all *P* <0.05). The cumulative incidence rates of HCC increased significantly in association with elevated LS value in 3 stratified groups (LS value <8, 8–13, and >13 kPa; log-rank test, *P* <0.001), and with higher histological fibrosis stage in 3 stratified groups (F0–2, F3, and F4; log-rank test, *P* <0.001). On multivariate analysis, along with age, LS value was an independent predictor of HCC development (hazard ratio 1.041, *P* <0.001), whereas histological staging was not (*P* >0.05).

TE predicted HCC development independently in patients with CHB starting antiviral therapy. However, further investigation is needed to determine whether the current surveillance strategy can be optimized based on the LS value at the time of starting antiviral therapy.

## INTRODUCTION

Chronic hepatitis B virus (HBV) infection is a major cause of liver cirrhosis and hepatocellular carcinoma (HCC) worldwide, with more than 350 million people affected.^1^ During the last several decades, the development of antiviral agents has been a major breakthrough in the treatment of chronic hepatitis B (CHB). Their use in CHB has prevented disease progression and reduced the risk of HCC development.^2,3^ Although first-generation nucleos (t)ides analogs such as lamivudine have encouraged resistant HBV strain, more effective and less resistance-prone antiviral agents, such as entecavir and tenofovir, have been available recently. These newer agents suppress HBV completely and promptly in CHB patients regardless of high baseline viral load or the presence of drug resistance.^4,5^ Nevertheless, a low, but clinically relevant risk of HCC development has still remained in CHB patients receiving antiviral therapy.^6^

In this era of potent antiviral therapy, the prognostic significance of serum HBV DNA level, which was considered a risk factor for HCC development, has substantially diminished. Thus, it can be hypothesized that fibrotic burden that is significantly related to the risk of developing HCC^7,8^ can stratify individual patients into different risks of developing HCC among CHB patients starting antiviral therapy. For evaluating the extent of liver fibrosis, liver biopsy (LB) remains the gold standard to date. However, due to the limitations of LB such as invasiveness, sampling error, and inter- and intraobserver variability, LB is often considered an “imperfect” surrogate marker for liver fibrosis.^9^ In addition, serial LBs are not feasible during antiviral therapy in clinical practice.^9^ Thus, to overcome the pitfalls of LB, noninvasive methods to assess liver fibrosis have been developed.

Transient elastography (TE) is one of the most widely validated noninvasive tools for assessing fibrotic burden.^10,11^ The prognostic value of liver stiffness (LS) as measured by TE in predicting critical events related to fibrosis progression, including the development of portal hypertension-related complications and HCC, has been well established in several longitudinal studies.^12–15^ Moreover, recent studies demonstrated that TE can predict HCC or liver-related events (LREs) even in patients receiving antiviral therapy.^16–18^ Although a recent study demonstrated that TE is more useful in predicting LREs when compared with histological data in CHB patients before starting antiviral therapy,^19^ further validation of TE in predicting HCC in the era of antiviral therapy is still required.

In this multicenter, retrospective study, we aimed to evaluate the prognostic value of TE in predicting risk of developing HCC as compared with the prognostic value of histological data in CHB patients starting antiviral therapy.

## METHODS

### Patients

From November 2005 to January 2015, 465 CHB patients who underwent LB and TE before starting antiviral therapy at 3 tertiary centers were considered for inclusion. The indication for LB was the assessment of the severity of liver fibrosis and inflammation before starting antiviral therapy. CHB was defined as the persistent presence of serum hepatitis B surface antigen for >6 months.^17^ Antiviral therapy was initiated in accordance with the treatment guidelines of the Korean Association for the Study of the Liver^20^ and reimbursement guidelines of the National Health Insurance Service in Korea. Virological response (VR) was defined as a reduction in HBV DNA levels to <2000 IU/mL.

Exclusion criteria were as follows: failure to obtain reliable LS values (valid shot = 0), an invalid LS value, delay between LB and TE>1 month, starting antiviral therapy more than 1 month after LB, presence of HCC at enrollment or history of it, HCC development within 6 month after enrollment, history of previous antiviral therapy, history of decompensated cirrhosis, Child–Pugh class B or C cirrhosis at enrollment, unsuitable quality of LB specimen for appropriate interpretation, coinfection with hepatitis C, hepatitis D, or HIV, right-sided heart failure, pregnancy, and loss to follow-up (Supplementary Figure 1).

The study protocol conformed with the ethical guidelines of the 1975 Declaration of Helsinki and was approved by the Institutional Review Board of Severance Hospital as well as other institutes. Written informed consents were not required due to the retrospective nature of this study.

### LB Examination

LB specimens were prepared with hematoxylin and eosin (H&E) and Masson trichrome. All liver tissue samples were evaluated by experienced pathologists at each center who was blinded to the clinical data. The degree of liver histology was evaluated semiquantitatively according to the Batts and Ludwig scoring system.^21^ Fibrosis was staged as follows: F0, no fibrosis; F1, portal fibrosis without septa; F2, portal fibrosis and a few septa; F3, numerous septa without cirrhosis; and F4, cirrhosis. The necro-inflammatory activity was graded as A0, none; A1, minimal; A2, mild; A3, moderate; and A4, severe activity.

### LS Measurement Using TE

TE was performed as previously reported,^15,22,23^ and LS value was expressed as kilopascal (kPa). In this study, only LS values with at least 10 validated measurements and a success rate of at least 60%, and an interquartile range (IQR) to median value ratio (IQR/M) of <0.3 were considered reliable.

### Baseline Work-Up and Follow-Up

The baseline visit for enrollment was defined as the visit during which LB was performed. At baseline and during follow-up, all patient data were evaluated based on ultrasonography and laboratory work-up, including alpha-fetoprotein every 3 or 6 months for screening of HCC and other portal hypertension-related complications. The diagnosis of HCC was established based on the guideline of the American Association for the Study of Liver Disease.^24^ LRE was defined as hepatic decompensation (variceal bleeding, ascites, spontaneous bacterial peritonitis, hepatic encephalopathy, or hepatorenal syndrome), HCC, and liver-related death. To avoid statistical repetition, we selected the earliest LRE if a given patient experienced different types of LRE at different time points.

### Statistical Analysis

Data are expressed as the median with range, the mean ± SD, or number (n, %) as appropriate. Differences among continuous and categorical variables were examined using Student *t* test (or the Mann–Whitney test) and the *χ*^2^ test (or Fisher exact test), respectively. To assess the accuracy of LS value in diagnosing histological bridging fibrosis and cirrhosis, the receiver operating characteristics (ROC) curve and its 95% confidence interval (CI) were calculated.^23^

The cumulative incidence rates of HCC and LRE were analyzed using the Kaplan–Meier method with comparison using the log-rank test. Times to HCC and LRE development were calculated from the date of enrollment to the date of development of HCC and the first LRE or the last follow-up. To identify independent risk factors for developing HCC and LRE, Cox proportional hazards regression analysis was used.^23^ Statistical analyses were performed using SPSS software (version 18.0; SPSS Inc., Chicago, IL). A *P* value <0.05 was considered statistically significant.

## RESULTS

### Patient Characteristics

After excluding 84 patients according to our exclusion criteria, a total of 381 patients were finally selected for the statistical analysis (Supplementary Figure 1). The baseline clinical characteristics of the study population are described in Table 1. The mean age was 44.1 years, and male sex was predominant (n = 251, 65.9%). The mean LS value and ALT level were 13.9 kPa and 132.6 IU/L, respectively. The histological fibrosis stages were F0–1 in 44 (11.5%) patients, F2 in 93 (24.4), F3 in 94 (24.7), and F4 in 150 (39.4).

**TABLE 1:**
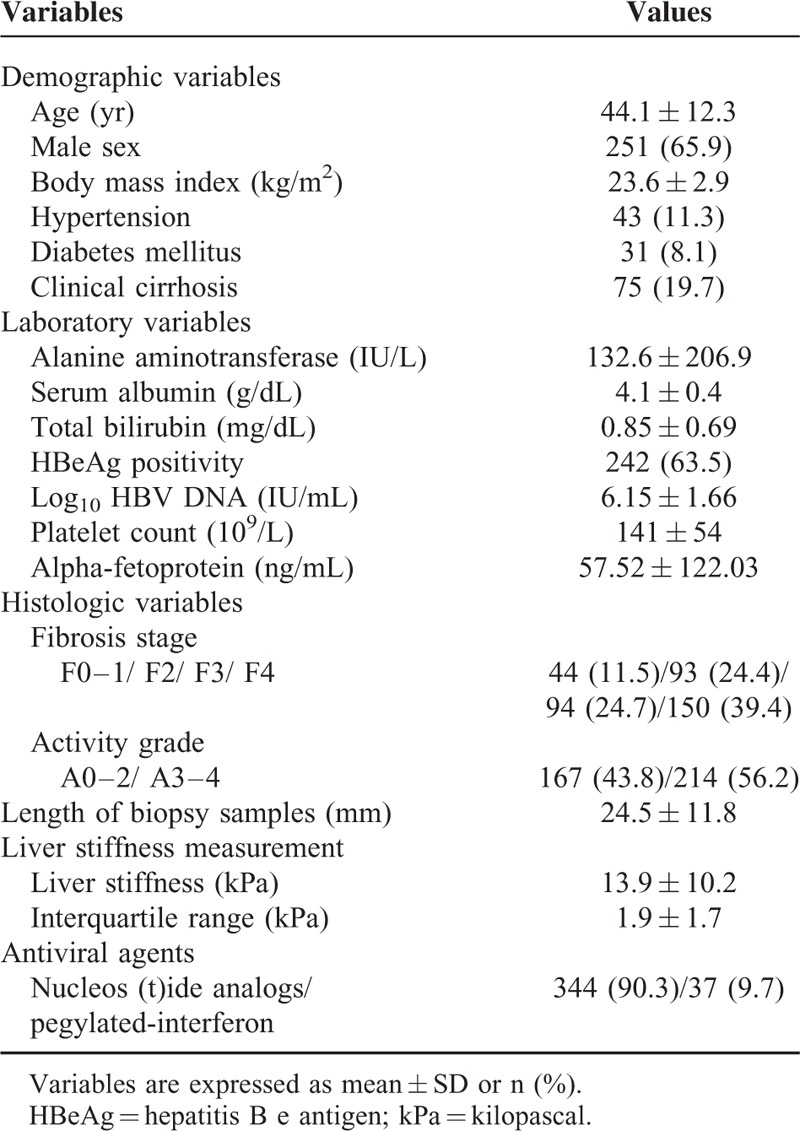
Baseline Characteristics of the Study Population (n = 381)

A total of 344 (90.3%) patients were treated with nucleos (t)ide analogs (67 with lamivudine, 21 with clevudine, 59 with telbivudine, 9 with adefovir, 10 with tenofovir, and 178 with entecavir), and 37 (9.7%) patients were treated with pegylated-interferon.

### Diagnostic Performances of TE and the Optimal LS Cutoff for Each Fibrosis Stage

The optimal cutoff LS values and corresponding diagnostic indexes of our study cohort were calculated (Supplementary Table 1). The AUCs of TE to predict ≥F2 (n = 337), ≥F3 (n = 244), and ≥F4 (n = 148) fibrosis stage were 0.804 (05% CI 0.747–0.962), 0.830 (95% CI 0.786–0.874), and 0.824 (95% CI 0.783–0.865), respectively. The cutoff LS values for ≥F2, ≥F3, and F4 were 7.5 kPa (sensitivity 79.5% specificity 65.9%), 9.5 kPa (sensitivity 76.6% specificity 80.3%), and 11.5 kPa (sensitivity 75.7% specificity 79.0%), respectively.

### HCC Development During Follow-Up

**FIGURE 1:**
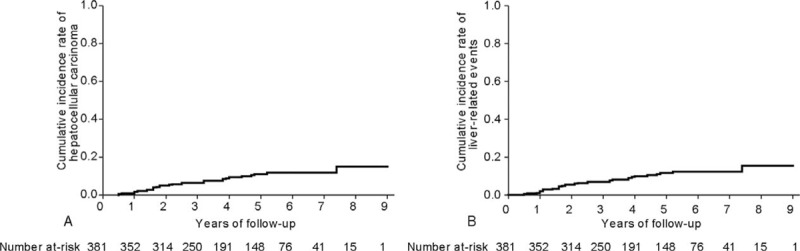
The cumulative incidence rates of HCC (A) and LRE (B) (Kaplan–Meier plot). The cumulative incidence rates of HCC at 3, 5, and 7 years were 6.5%, 11.2%, and 11.9%, respectively, with an annual incidence of 22 per 1000 person-years. The cumulative incidence rates of LREs at 3, 5, and 7 years were 7.0%, 11.7%, and 12.5%, respectively, with an annual incidence of 23 per 1000 person-years. HCC = hepatocellular carcinoma; LRE = liver-related event.

During a median follow-up period of 48.1 (IQR 30.3–69.3) months, 34 (8.9%) patients experienced HCC development. Furthermore, 36 (9.4%) patients experienced LRE development (31 HCCs and 5 hepatic decompensations as the first event). The cumulative incidence rates of HCC at 3, 5, and 7 years were 6.5%, 11.2%, and 11.9%, respectively, with an annual incidence of 22 per 1000 person-years (Figure 1A). The cumulative incidence rates of LRE at 3, 5, and 7 years were 7.0%, 11.7%, 12.5%, respectively, with an annual incidence of 23 per 1000 person-years (Figure 1B).

### Comparison of Baseline Characteristics of Patients With and Without HCC and LRE

When the baseline characteristics of patients with and without HCC were compared (Table 2), age (mean 54.5 vs. 43.0 years), proportion of diabetes mellitus (20.6 vs. 6.9%), histological F4 fibrosis stage (70.6 vs. 35.7%), and LS value (mean 20.0 vs. 13.3 kPa) were significantly higher in patients with HCC development than in those without (all *P* <0.05), whereas ALT level (52.4 vs. 140.5 IU/L) and platelet count (128 vs. 142 × 10^9^/L) were significantly lower in patients with HCC development than in those without (all *P* <0.05).

**TABLE 2:**
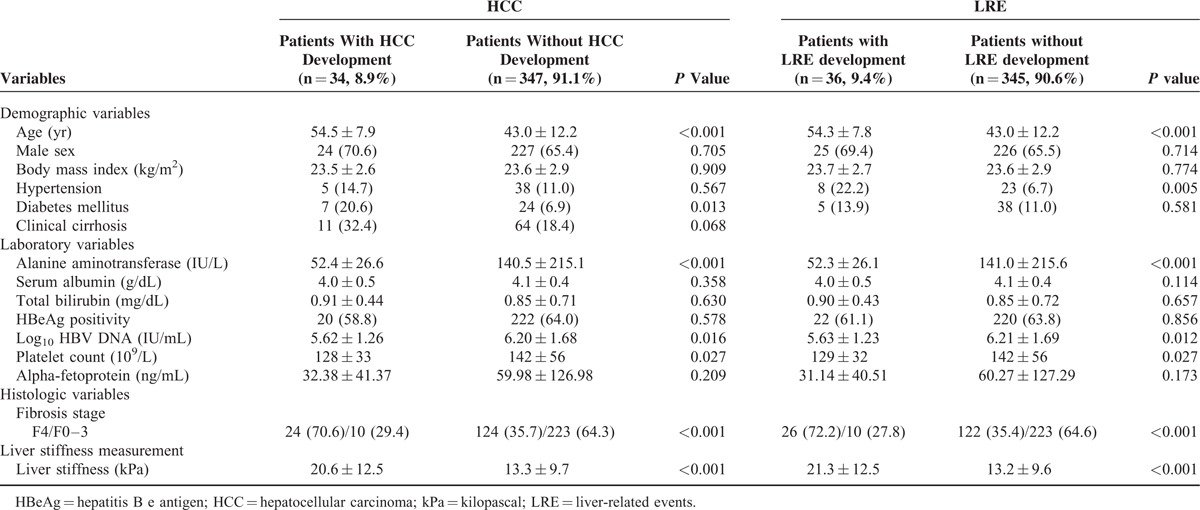
Comparison Between Patients With and Without HCC or LRE Development

When the baseline characteristics of patients who experienced an LRE were compared with those of patients who did not (Table 2), age (mean 54.3 vs. 43.0 years), proportion of hypertension (22.2 vs. 6.7%), histological F4 fibrosis stage (72.2 vs. 35.4%), and LS value (21.3 vs. 13.2 kPa) were significantly higher in patients with LRE development than in those without (all *P* <0.05), whereas ALT level (52.3 vs. 141 IU/L), HBV-DNA level (5.63 vs. 6.21 log_10_ IU/mL), and platelet count (129 vs. 142 10^9^/L) were significantly lower in patients with LRE development (all *P* <0.05).

### Comparison of the Prognostic Performance of LS Value and Histological Fibrosis Stage

The AUCs of the LS value and histological fibrosis stage for HCC development at 3- and 5- years are shown in Table 3. Although the AUC values of LS value to predict HCC development at 3-years and 5-years were higher than those of histological fibrosis stage, statistical significances were not reached (all *P* >0.05). Similar results were obtained with respect to LRE development (Table 3).

**TABLE 3:**
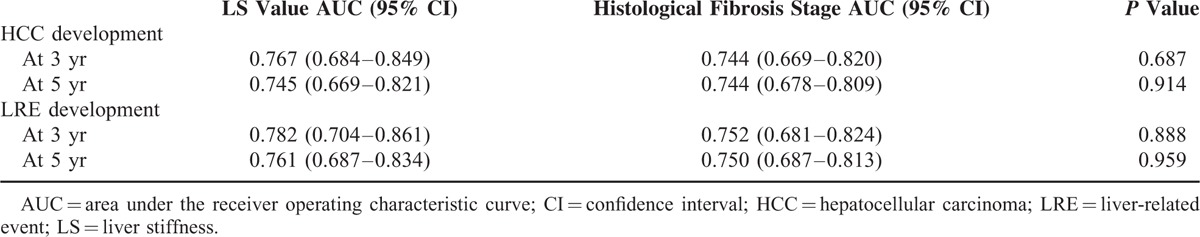
Comparison of the Prognostic Performance of LS Value and Histological Fibrosis Stage

### Independent Predictors of HCC and LRE Development

Univariate and subsequent multivariate analysis to identify independent predictors of HCC and LRE development are described in Table 4. On univariate analysis, age, diabetes mellitus, clinical cirrhosis, ALT level, fibrosis stage (F4 vs. F0–3), and LS values significantly predicted HCC development (all *P* <0.05), whereas age, diabetes mellitus, ALT level, serum albumin level, alpha-fetoprotein, fibrosis stage (F4 vs. F0–3), and LS values significantly predicted LRE development (all *P* <0.05). On subsequent multivariate analyses, LS value was an independent predictor of HCC development (*P* <0.001; adjusted HR 1.042, 95% CI 1.016–1.070) and LRE development (*P* = 0.006; adjusted HR 1.041, 95% CI 1.012–1.071), together with age (Table 4), whereas histological fibrosis stage was not an independent predictor for either HCC or LRE development (all *P* >0.05). When LS value and histological fibrosis stage were separately entered into multivariate analyses to prevent statistical collinearity, LS value was selected as one of the independent predictors of HCC (HR 1.058, 95% CI 1.020–1.097, *P* = 0.002) and LRE development (HR 1.052, 95% CI 1.018–1.087, *P* = 0.003) (Supplementary Table 2, whereas histological fibrosis stage was not (*P* = 0.089 for HCC, *P* = 0.095 for LRE) (Supplementary Table 3).

**TABLE 4:**
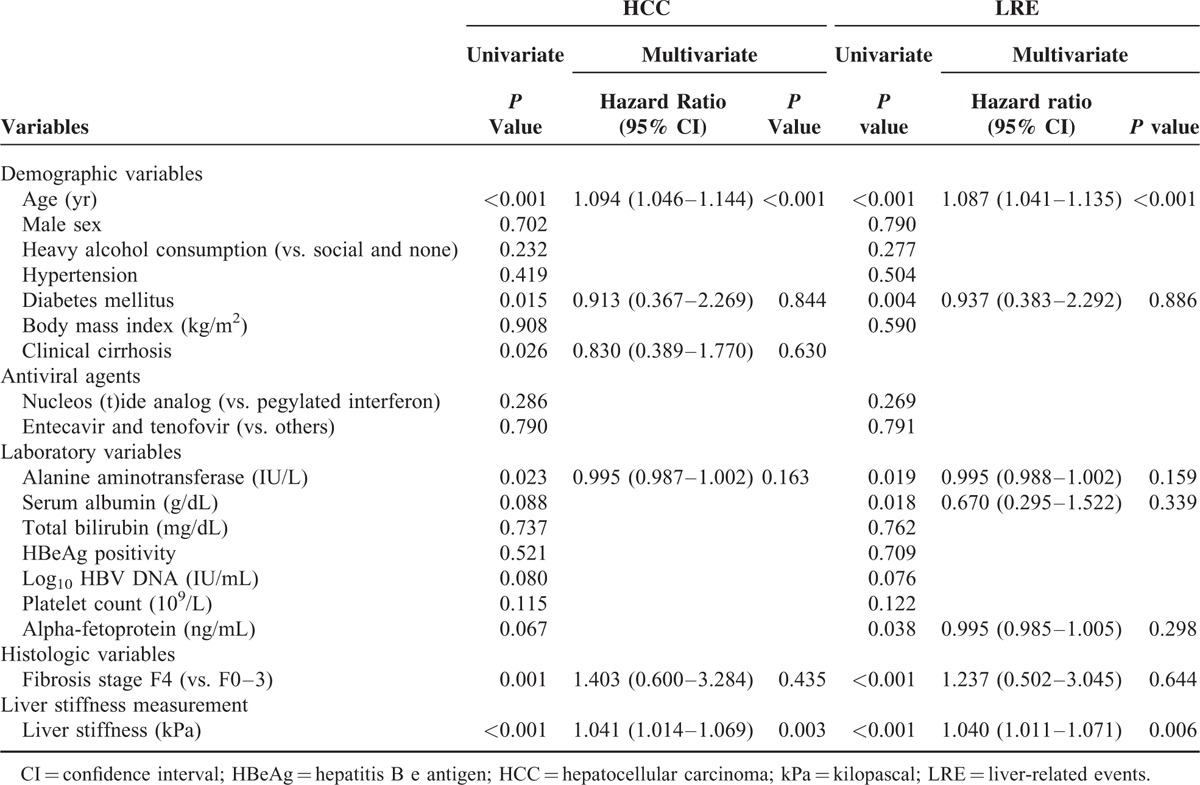
Independent Predictors of HCC or LRE Development

**FIGURE 2:**
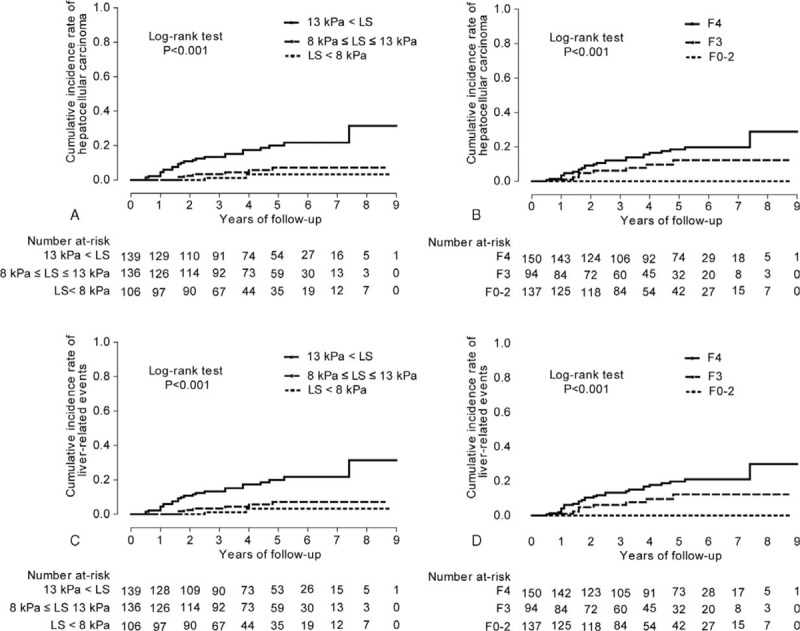
The cumulative incidence rates of HCC based on stratified LS values (<8, 8–13, and >13 kPa) and histological fibrosis stage (F0–2, F3, and F4) (A and B), and the cumulative incidence rates of LRE based on stratified LS values and histological fibrosis stage (C and D). The cumulative incidence rates of HCC and LRE increased significantly in association with higher LS value and with higher histological fibrosis stage (log-rank test, all *P* <0.001). HCC = hepatocellular carcinoma; LRE = liver-related event.

Considering the independent prognostic significance of LS values, we stratified our study population into 3 groups using the cutoff LS values of 8.0 and 13 kPa according to the stratifications used in several previous studies.^15,17^ The risk of HCC and the risk of LRE development increased in association with higher LS values among 3 stratified groups (log-rank test, all *P* <0.001) (Figure 2A and B). Although histological fibrosis stage was not a significant predictor of HCC and LRE development, when the patients were divided into 3 groups according to histological fibrosis stage (F0-2, F3, and F4), the risk of HCC and the risk of LRE development also increased in association with higher histological fibrosis stage (log-rank test, all *P* <0.001) (Figure 2C and D).

### Association Between the Change in LS Values and the Risk of Developing HCC or LRE

**FIGURE 3:**
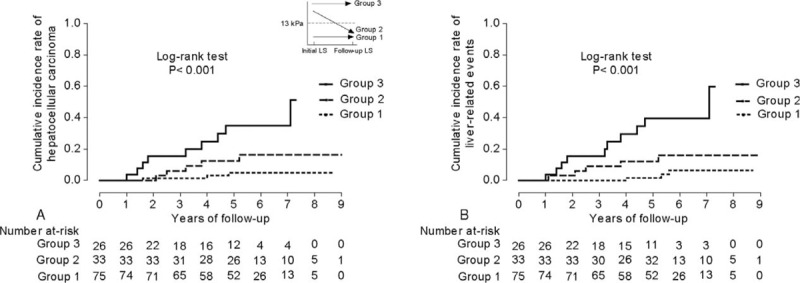
The cumulative incidence rates of HCC (A) and LRE (B) according to the changes in LS values (n = 134). The overall incidence rates of HCC and LRE differed significantly among the 3 groups (log-rank test, all *P* <0.001). HCC = hepatocellular carcinoma; LRE = liver-related event.

Of the study population, a second TE examination was available at the time of VR after a median of 13.2 (range, 6.0–24.0) months in 139 (36.5%) patients. We excluded 5 patients who showed an increase in LS value from <13 kPa at baseline to ≥13 kPa at follow-up to prevent statistical error caused by a small sample size. The remaining patients were divided into 3 groups (group 1: n = 75, LS value <13 kPa at baseline and follow-up; group 2: n = 33, LS value ≥13 kPa at baseline and <13 kPa at follow-up; group 3: n = 26, LS values >13 kPa both at baseline and follow-up). The cumulative incidence rates of HCC and LRE differed significantly among the 3 groups (log-rank test, all *P* <0.001) (Figure 3A and B).

## DISCUSSION AND CONCLUSIONS

In this study, we aimed to evaluate whether TE, when compared with histological data, can predict the risk of HCC and LRE development in CHB patients starting antiviral therapy. To our knowledge, this is the first Asian longitudinal study with a relatively large sample size demonstrating that the prognostic value of TE may be better than that of histological information in assessing the risk of HCC and LRE development. Our study also demonstrated that changes in LS values can monitor the changing risk of HCC or LRE development during antiviral therapy.

The results of our study address several important clinical points. First, LB, as a gold standard, provide accurate information regarding the degree of liver fibrosis and necroinflammation, enable clinicians in determining whether to start antiviral therapy, and provide prognostic information.^9^ Indeed, as shown in our study, histological assessment predicted the risk of HCC and LRE development at 3- and 5-years of antiviral therapy with acceptable accuracy (AUC around 0.75), which was statistically similar to that of LS value. However, histological assessment was not selected as an independent predictor of HCC and LRE development when adjusted with and without LS value. In contrast, it was demonstrated that TE could assess the fibrotic burden with acceptable accuracy (AUC more than 0.8 for each fibrosis stage) similar to a vast amount of previous studies,^13,19,25^ and that TE was independently prognostic in predicting HCC and LRE development even after adjusting for LB and other variables. These findings support the necessity to check LS values at the time of starting antiviral therapy. Our findings also supported the use of TE to assess the changing risk of HCC and LRE development. Because serial LBs are not feasible in real clinical practice due to the inherent invasiveness of biopsy, TE can be used to monitor the changes in LS values during antiviral therapy.

Second, our study showed that the risk of developing HCC or experiencing an LRE remained significant with cumulative incidence rates of 11.2% and 11.7% at 5 year, respectively, although it has been known that the risks significantly decreased due to appropriate antiviral therapy.^26,27^ This supports the need for risk prediction to assist prognostication and HCC surveillance even with antiviral therapy. Although HBV DNA level was one of the most important predictors of HCC development in the era prior to antiviral therapy,^28,29^ the preexisting or remained fibrotic burden after antiviral therapy has recently received the attention as a significant prognostic candidate due to diminished prognostic significance of HBV DNA by active and potent antiviral therapy.^7^ In this regard, in the era of antiviral therapy, the LS value, which can be used to assess preexisting or residual fibrotic burden, may be a more appropriate risk prediction tool for HCC development. Accordingly, recent studies demonstrated that the LS-based HCC risk scores had better predictive performance than conventional scores in CHB patients receiving antiviral therapy.^17,30^

In our study, a 1-point increase in LS value was associated with a 1.041- and 1.040-fold increase in the risk of HCC and LRE development, respectively. These results emphasize the prognostic significance of LS values in determining the risk of developing HCC in the era of antiviral therapy and are consistent with the results of several previous studies with similar clinical settings.^18,19,31^ A previous study by Kim et al^19^ demonstrated that LS value was an independent predictor of LRE development, whereas histological fibrosis stage was not, in patients showing histologically advanced liver fibrosis and starting nucleot (s)ide analogs. Another study by Lee et al demonstrated that LS values at complete VR are useful for predicting LRE development in patients receiving entecavir.^17^ When we divided our study population into 3 groups using the stratified LS value similar to the study by Lee et al, the risk of developing HCC significantly increased in the groups with high LS values. Similar to our study, prior studies have shown that LS values significantly decreased in most CHB patients during antiviral therapy, and those changes may reflect the changing risk of HCC or LRE development during antiviral therapy.^17,24,32^

Several issues still remained unresolved in our study. First, the number of patients who developed HCC was small (8.9%), which was related to the characteristics of our study population receiving antiviral therapy. Nevertheless, the homogeneity of our population with respect to antiviral treatment, histological data, and follow-up duration may have played a role in revealing the clinical significance of TE. Second, if histological data had been available at the time of VR, the association of LS change with histological fibrosis change might have been more clearly determined. A well-designed future study with follow-up histological data is required to confirm whether TE can monitor the changes in the fibrotic burden during and after antiviral therapy. Finally, after a rigorous review of the literature, we decided to focus on the performance of TE instead of simple noninvasive methods such as AST to platelet ratio index (APRI), FIB-4, and Forn index. Despite being a good alternative to LB, TE is not widely available due to its high cost, especially in resource-limited countries. Thus, future studies should focus on simple noninvasive methods.

In conclusion, our study showed the predictive value of LS measurement using TE, as compared with the histological fibrosis stage, in predicting HCC and LRE development in patients receiving antiviral therapy. Despite the reduction in HCC and LRE risk attributable to effective antiviral therapy, the risk remains substantial. Our study supports that tailored surveillance strategies for HCC could be established based on LS values. However, further investigation is needed to determine whether the current surveillance strategy can be optimized based on the LS value at the time of starting antiviral therapy.

## Supplementary Material

Supplemental Digital Content
