# Risk Assessment of Hepatocellular Carcinoma Using Transient Elastography Vs. Liver Biopsy in Chronic Hepatitis B Patients Receiving Antiviral Therapy: Erratum

**DOI:** 10.1097/01.md.0000488768.90771.b8

**Published:** 2016-07-01

In the article “Risk Assessment of Hepatocellular Carcinoma Using Transient Elastography Vs. Liver Biopsy in Chronic Hepatitis B Patients Receiving Antiviral Therapy”,^1^ which appeared in Volume 95, Issue 12 of *Medicine*, Drs. Yeon Seok Seo and Mi Na Kim contributed equally as first authors. The article has since been corrected online.
