# Supplementary material for: Risk Assessment of Hepatocellular Carcinoma Using Transient Elastography Vs. Liver Biopsy in Chronic Hepatitis B Patients Receiving Antiviral Therapy
Source: Medicine (Baltimore). 2016 Mar 25;95(12):e2985. doi: 10.1097/MD.0000000000002985 (PMC4998368; doi:10.1097/MD.0000000000002985)

Supplementary table 1. Diagnostic performances of TE and the optimal LS cutoff values for each fibrosis stage

**Variables**	**≥F2**	**≥F3**	**F4**
Number of patients, n (%)	337	244	148
AUC	0.804	0.830	0.824
95% CI	0.747 - 0.862	0.786 - 0.874	0.783 - 0.865
P value	<0.001	<0.001	<0.001
Cutoff value (kPa)	7.5	9.5	11.5
Sensitivity (%)	79.5	76.6	75.7
Specificity (%)	65.9	80.3	79.0

TE, transient elastography; LS, liver stiffness; AUC, area under the receiver operating characteristic curve; CI, confidence interval; kPa, kilopascal.

Supplementary table 2. Independent risk factors of HCC development

Variables	Hepatocellular carc		
	Univariate	Multivariate using liver stiffness	
	***P* value**	**Hazard ratio (95% CI)**	***P* value**
Demographic variables			
Age (years)	<0.001	1.156 (1.076-1.242)	<0.001
Male gender	0.702		
Heavy alcohol consumption (vs. social and none)	0.232		
Hypertension	0.419		
Diabetes mellitus	0.015	0.593 (0.160-2.196)	0.434
Body mass index (kg/m^2)	0.908		
Clinical cirrhosis	0.026	0.690 (0.251-1.893)	0.471
Antiviral agents			
Nucleos(t)ide analog (vs. Peglyated interferon)	0.286		
Entecavir and tenofovir (vs. others)	0.790		
Laboratory variables			
Alanine aminotransferase (IU/L)	0.023	1.013 (0.969-1.059)	0.577
Serum albumin (g/dL)	0.088		
Total bilirubin (mg/dL)	0.737		
HBeAg positivity	0.521		
log HBV DNA (IU/mL)	0.080		
Platelet count (10^9/L)	0.115		
Alpha-fetoprotein (ng/mL)	0.067		
Histologic variables			
Fibrosis stage F4 (vs. F0-3)	0.001		
Liver stiffness measurement			
Liver stiffness (kPa)	<0.001	1.058 (1.020-1.097)	0.002

HCC, hepatocellular carcinoma; LRE, liver-related events; CI, confidence interval; HBV, hepatitis B virus; HBeAg, hepatitis I

Supplementary table 3. Independent risk factors of LRE development

Variables	LRE		
	Univariate	Multivariate using liver stiffness	
	P value	Hazard ratio (95% CI)	*P* value
Demographic variables			
Age (years)	<0.001	1.137 (1.062-1.216)	<0.001
Male gender	0.79		
Heavy alcohol consumption (vs. social and none)	0.277		
Hypertension	0.504		
Diabetes mellitus	0.004	0.855 (0.266-2.747)	0.793
Body mass index (kg/m^2)	0.59		
Clinical cirrhosis		0.853 (0.330-2.210)	0.744
Antiviral agents			
Nucleos(t)ide analog (vs. Peglyated interferon)	0.269		
Entecavir and tenofovir (vs. others)	0.791		
Laboratory variables			
Alanine aminotransferase (IU/L)	0.019	1.010 (0.967-1.054)	0.666
Serum albumin (g/dL)	0.018		
Total bilirubin (mg/dL)	0.762		
HBeAg positivity	0.709		
log HBV DNA (IU/mL)	0.076		
Platelet count (10^9/L)	0.122		
Alpha-fetoprotein (ng/mL)	0.038		
Histologic variables			
Fibrosis stage F4 (vs. F0-3)	<0.001		
Liver stiffness measurement			
Liver stiffness (kPa)	<0.001	1.052 (1.018-1.087)	0.003

HCC, hepatocellular carcinoma; LRE, liver-related events; CI, confidence interval; HBV, hepatitis B virus; HBeAg, hepatitis I

Supplementary figure 1 Flow of selecting the study population. A total of 465 patients with CHB were initially recruited. Of these, 84 patients were excluded according to our exclusion criteria. Finally, 381 patients were selected for statistical analysis. CHB, chronic hepatitis B.

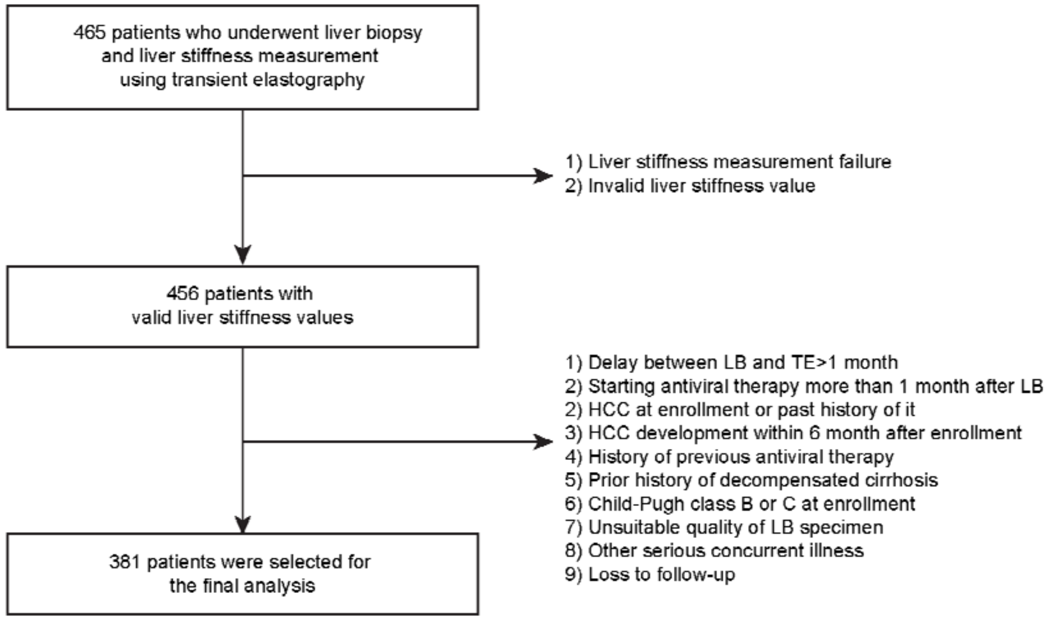

Supplement: Supplemental Digital Content [file medi-95-e2985-s001.pdf]
